# Steatotic liver disease interacts with a polygenic risk score for triglyceride clearance to impact the risk of hypertriglyceridaemia: The Maastricht Study

**DOI:** 10.1007/s00125-025-06479-3

**Published:** 2025-06-27

**Authors:** Zhewen Ren, Anke Wesselius, M. Eline Kooi, Marleen van Greevenbroek, Pieter Dagnelie, Tos T. J. M. Berendschot, Abraham A. Kroon, Alfons J. H. M. Houben, Coen D. A. Stehouwer, Martijn C. G. J. Brouwers

**Affiliations:** 1 Department of Internal Medicine, Maastricht University Medical Center, Maastricht, the Netherlands; 2 Cardiovascular Research Institute Maastricht (CARIM), Maastricht University, Maastricht, the Netherlands; 3 Laboratory for Metabolism and Vascular Medicine, Maastricht University, Maastricht, the Netherlands; 4 Department of Epidemiology, Maastricht University, Maastricht, the Netherlands; 5 Institute of Nutrition and Translational Research in Metabolism (NUTRIM), Maastricht University, Maastricht, the Netherlands; 6 Department of Radiology and Nuclear Medicine, Maastricht University Medical Center, Maastricht, the Netherlands; 7 University Eye Clinic Maastricht, Maastricht University Medical Center, Maastricht, the Netherlands; 8 Department of Chronic Diseases and Metabolism (CHROMETA), KU Leuven, Leuven, Belgium; 9 Care and Public Health Research Institute (CAPHRI), Maastricht University, Maastricht, the Netherlands

**Keywords:** Hypertriglyceridaemia, Interaction, Polygenic risk score, Steatotic liver disease

## Abstract

**Aims/hypothesis:**

The pathogenesis of hypertriglyceridaemia is explained by a complex interplay between genetic and environmental factors. We hypothesised that intrahepatic lipid (IHL) content, which drives the production of triacylglycerol-rich VLDL particles, interacts with a polygenic risk score (PRS) for triglyceride clearance to impact the risk of hypertriglyceridaemia.

**Methods:**

We used data from The Maastricht Study, a population-based prospective cohort study (*n*=3810; age: 60 years, 48% women, 10% hypertriglyceridaemia, 26% steatotic liver disease). We performed multivariable linear regression analyses to assess the impact of the cross-sectional interaction between IHL content (quantified by MRI) and a PRS for triglyceride clearance (based on nine SNPs) on fasting serum triglycerides, after adjustment for sociodemographic, lifestyle and cardiovascular risk factors. We subsequently explored whether a similar longitudinal interaction affects incident CVD during a 10 year follow-up.

**Results:**

There was an impact of interaction between IHL content and the PRS for triglyceride clearance on serum triglycerides (*p*=0.005). The strength of the association between a high PRS and risk of hypertriglyceridaemia was larger in individuals with steatotic liver disease (OR 6.196; 95% CI 3.966, 9.768) than in those without (OR 1.618; 95% CI 1.110, 2.380). A similar trend was observed for incident CVD risk (*p*=0.078).

**Conclusions/interpretation:**

Genetically predisposed individuals have a substantially higher risk of hypertriglyceridaemia when they also have steatotic liver disease. This gene–environment interaction might contribute to more personalised treatment approaches, which require further exploration in future studies.

**Graphical Abstract:**

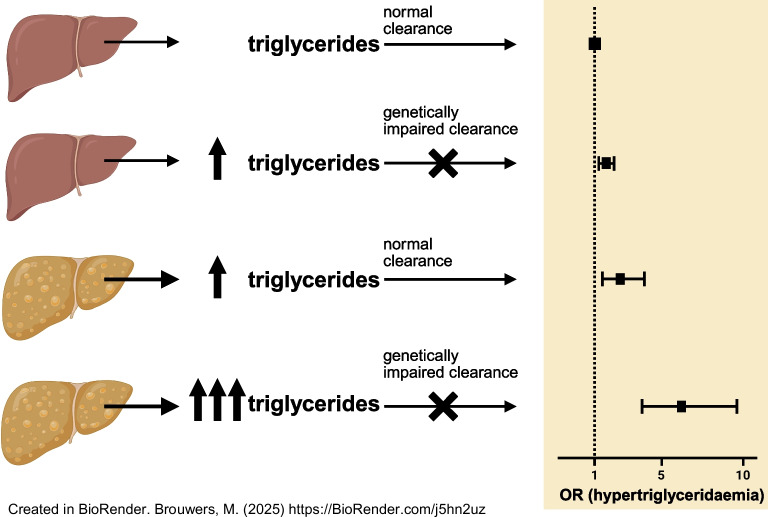

**Supplementary Information:**

The online version of this article  (10.1007/s00125-025-06479-3) contains peer-reviewed but unedited supplementary material.


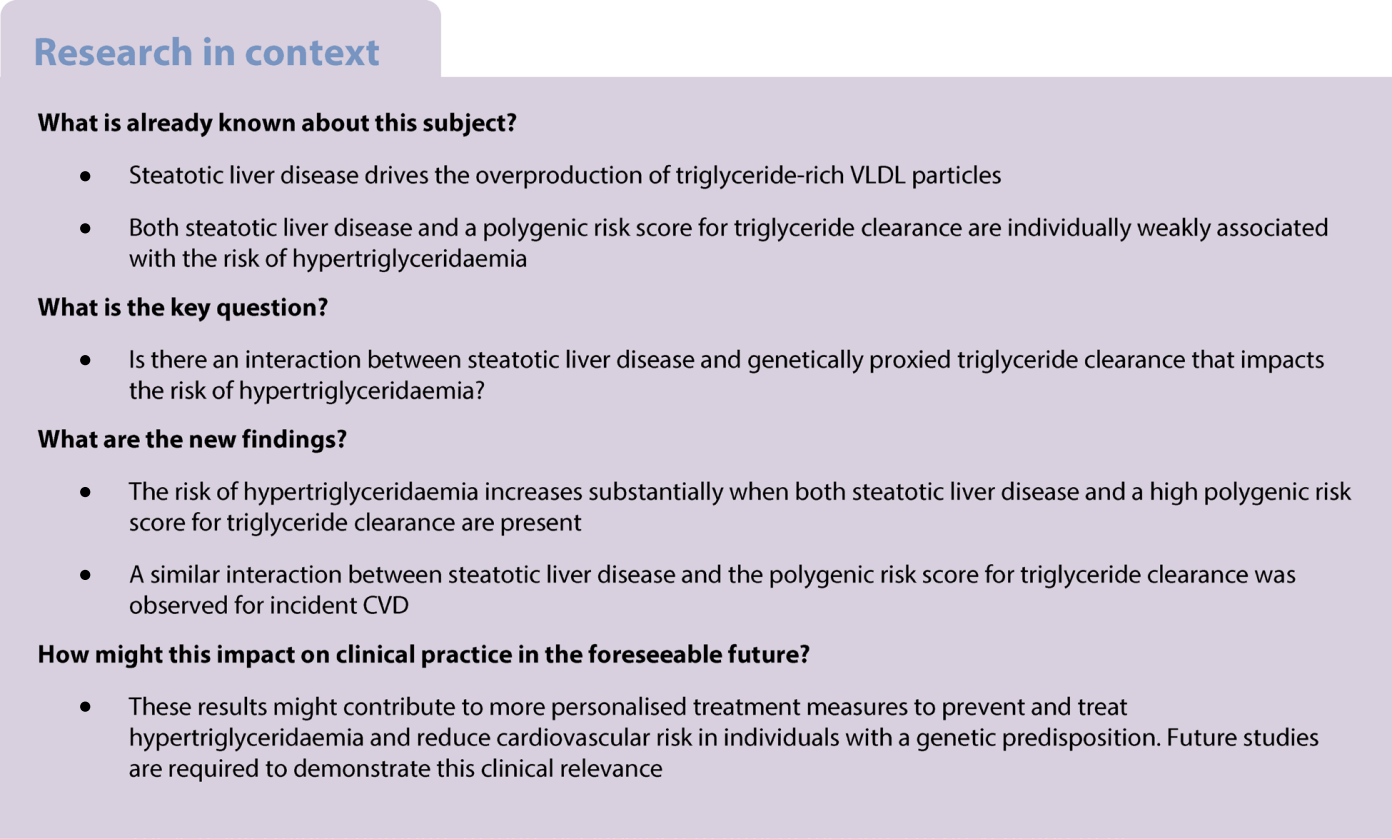


## Introduction

Hypertriglyceridaemia, a well-reported risk factor for CVD [1, 2], is very common in individuals with type 2 diabetes [3–5]. Over the past years, a vast number of genes have been identified that collectively contribute to an increased risk of hypertriglyceridaemia [6–8]. Nevertheless, genetic factors alone are not sufficient to predict hypertriglyceridaemia. Hegele and colleagues have argued that the pathogenesis of hypertriglyceridaemia is explained by a complex interplay between multiple primary (genetic) and secondary factors [9].

Metabolic dysfunction-associated steatotic liver disease (MASLD), previously referred to as non-alcoholic fatty liver disease (NAFLD) [10], is highly prevalent in Western society (estimated prevalence ~30% [11, 12]). The high prevalence is explained by an unhealthy lifestyle, i.e. a high energy intake [13], high intake of nutrients that promote intrahepatic lipid (IHL) accumulation (fructose and saturated fatty acids [12, 14, 15]) and a sedentary lifestyle [16]. MASLD has previously emerged as a cardiovascular risk factor [17]. We recently applied a Mendelian randomisation approach to show that MASLD appears to play a causal role in the pathogenesis of CVD [18], which, at least in part, is mediated by serum lipids [19, 20]. Stable isotope studies have shown that IHL accumulation drives the overproduction of triacylglycerol-rich VLDL particles by the liver [21–23], contributing to an increased risk of hypertriglyceridaemia.

**Fig. 1:**
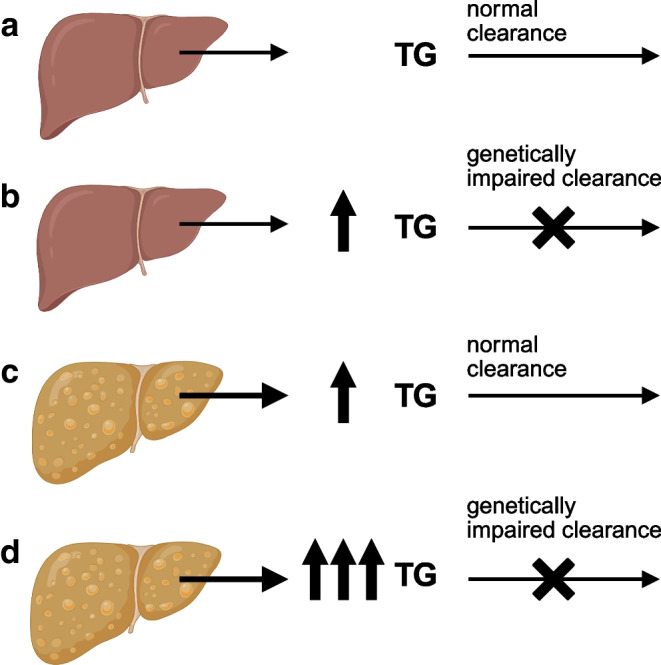
Hypothesised interaction between steatotic liver disease and genetically impaired triglyceride clearance and effect on serum triglycerides. It is anticipated that a genetic impairment of triglyceride clearance or steatotic liver disease will cause a moderate increase in serum triglycerides (**b** and **c**, respectively), whereas the combination of both will have a synergistic effect (**d**), as compared with a situation in which triglyceride clearance is not genetically impaired and steatotic liver disease is absent (**a**). Created in BioRender. Brouwers, M. (2025) https://BioRender.com/gz5qkgr. TG, triglyceride

Based on this, we hypothesised that steatotic liver disease may be a secondary factor that interacts with susceptibility genes to impact the risk of hypertriglyceridaemia. More specifically, we hypothesised that individuals with a high IHL content are at a particularly high risk of hypertriglyceridaemia (and consequently of CVD) when the lipid particles that are overproduced are not effectively cleared due to a primary defect in genes affecting both triglyceride hydrolysis and receptor-mediated clearance of remnant particles (Fig. 1).

Therefore, the aim of this study is to investigate the impact of interaction between IHL content and a polygenic risk score (PRS) for triglyceride clearance on the risk of hypertriglyceridaemia. Moreover, we explored whether the same interaction affects incident CVD risk during a 10 year follow-up.

## Methods

### Study population

We used data from The Maastricht Study, a prospective cohort study enriched with individuals with type 2 diabetes [24]. In brief, this study focuses on the aetiology, pathophysiology, complications and comorbidities of type 2 diabetes and is characterised by an extensive phenotyping approach. Eligible for participation were all individuals aged between 40 and 75 years and living in (and in the surrounding areas of) Maastricht, the Netherlands. Participants were recruited through mass media campaigns, the municipal registries and the regional Diabetes Patient Registry via mailings. Recruitment was stratified according to known type 2 diabetes status, with an oversampling of individuals with type 2 diabetes. The present study includes data from participants who completed the baseline measurements from November 2010 until October 2020 and had undergone valid MRI measurements of the liver (*n*=5021 participants). Of these, 1004 (19.9%) had missing data on genotyping, resulting in a study population of 4017 participants. We further excluded 78 (1.5%) participants with implausible energy intake (men: <3.3 MJ (800 kcal) or >17.6 MJ (4200 kcal) per day; women: <2.1 MJ (500 kcal) or >14.6 MJ (3500 kcal) per day) and 129 (2.5%) participants with missing data on alcohol intake, resulting in a final study population of 3810 participants (See Electronic supplementary material [ESM] Fig. 1). Missing data were not imputed. The Maastricht study has been approved by the institutional medical ethics committee (NL31329.068.10) and the Minister of Health, Welfare and Sport of the Netherlands (permit 131088-105234-PG). All participants gave written informed consent.

### Assessment of IHL content

IHL content was assessed through Dixon MRI using a 3.0 Tesla MRI system (MAGNETOM Prismafit, Siemens Healthineers, Erlangen, Germany) with body matrix and supine radiofrequency coils, as described in detail elsewhere [25].

This method was validated and calibrated against proton magnetic resonance spectroscopy (^1^H-MRS), the gold standard to non-invasively quantify IHL content, in 36 participants. After calibration, the intra-class correlation coefficient between Dixon MRI and ^1^H-MRS was 0.989 (95% CI 0.979, 0.994) [25].

Steatotic liver disease was defined by the presence of hepatic steatosis with an IHL content ≥5.56% [26]. The cutoff value for IHL content, originally expressed as CH_2_ (H_2_O + CH_2_), corresponds to 5.89% when IHL content is expressed as CH_2_/H_2_O, as was done in the current study [14].

### PRS for triglyceride clearance

Genotyping was done with the use of the Illumina Global Screening Array BeadChip (Infinium iSelect 24×1 HTS Custom BeadChip Kit) at the Human Genotyping Facility of the Genetic Laboratory of the Department of Internal Medicine at Erasmus MC Rotterdam, the Netherlands. Details concerning the processes of quality control and imputation are described in detail elsewhere [27].

Genetic variants associated with triglyceride clearance were retrieved from a large-scale genome-wide association study (GWAS) for serum lipids among 312,571 participants of various ethnicities [28]. This study reported 151 SNPs associated with serum triglycerides (*p*<5 × 10^−8^) after adjustment for age, sex and the first ten principal components of ancestry. Among them, nine SNPs were excluded due to being monoallelic in Europeans. Based on GeneCards and PubMed searches, the remaining 142 genes were subsequently categorised as: (1) involved in triglyceride clearance only (*n*=9); (2) involved in triglyceride production only (*n*=12); (3) involved in triglyceride production and clearance (*n*=5); (4) uncertain function (*n*=8); and (5) no results (*n*=108) (ESM Table 1, ESM Methods). We used the genes in the first group for our primary analyses. To ensure the efficiency of the PRS, several criteria were applied to filter out redundant variants: (1) SNPs with a minor allele frequency lower than 0.01; (2) SNPs in linkage disequilibrium (LD; *r*^2^≥0.1); (3) mismatching SNPs (e.g. a SNP with A/C in the previous GWAS and G/T in The Maastricht Study); (4) SNPs with low imputation quality (imputation score [INFO] <0.8). The nine SNPs were all eligible for this study and are presented in Table 1.

**Table 1:** List of serum triglyceride-associated genes involved in triglyceride clearance only

SNP	Chr:Pos	EA	NEA	EAF	β	SE	Nominated gene^a^
rs67461605	1:63070537	D	I	0.3573	−0.0756	0.0027	(*ANGPTL3*)
rs1077835	15:58723426	A	G	0.6836	−0.021	0.003	*LIPC*
rs10846744	12:125312425	C	G	0.3084	0.0252	0.0033	*SCARB1*
rs116843064	19:8429323	A	G	0.0193	−0.2524	0.0102	*ANGPTL4*
rs1569209	8:19830170	T	G	0.9206	0.2112	0.0053	(*LPL*)
rs1788783	18:21161134	T	C	0.504	−0.0158	0.0027	*NPC1*
rs1801689	17:64210580	A	C	0.969	0.0801	0.0081	*APOH*
rs2281721	1:230297136	T	C	0.5522	−0.0377	0.0028	*GALNT2*
rs34078567	19:45413224	D	I	0.6105	0.065	0.0029	(*APOC1*)

^a^Genes for variants that are outside the transcript boundary of a protein-coding gene are shown with the nearest gene in parentheses

Effect allele (EA) and non-effect allele (NAE) refer to DNA nucleotides (A, T, C, G) or insertions (I) or deletions (D)

Chr, chromosome; Pos, position

The PRS for triglyceride clearance was calculated by multiplying the dosage of effect allele for each SNP by its corresponding weight (effect size) and then summing all variants together. The effect size for each variant was derived from the previous GWAS [28] (ESM Methods). Effect allele dosage of a SNP for each participant was coded as 0, 1 and 2 for non-carriers, heterozygous carriers and homozygous carriers, respectively. For imputed data, genotype dosage data were converted to best guess genotypes based on a minimum genotype probability of *p*>0.8 [27].

### Measurement of serum triglycerides

Blood was drawn after an overnight fast. Serum triglycerides were measured by use of an automatic analyser (Beckman Synchron LX20, Beckman Coulter, Brea, USA) in venous blood samples. A detailed description of protocols for the laboratory assessments has been reported elsewhere [24]. Hypertriglyceridaemia was defined as serum triglycerides ≥2.3 mmol/l [29].

### Diagnosis of CVD at baseline and during follow-up

CVD status was assessed using a self-reported questionnaire both at baseline and annually during follow-up over 10 years [24]. CVD was defined as: (1) myocardial infarction; (2) cerebrovascular infarction and/or haemorrhage; (3) percutaneous artery angioplasty or vascular surgery of the coronary, abdominal, peripheral or carotid arteries. Of the 3301 participants free from CVD at baseline, 3217 had valid annual follow-up data on incident CVD (ESM Fig. 2).

### Covariates and other variables

Age, sex, education status (low, medium, high) and smoking status (never, former or current smoker) were assessed from questionnaires [24]. Alcohol consumption was assessed by a tailor-made food frequency questionnaire (FFQ) [30]. Medication use was assessed during medication interviews. Blood pressure was measured as the mean of at least three blood pressure readings (Omron 705IT, Japan).

Participants underwent a standardised 2 h 75 g OGTT after an overnight fast to determine glucose metabolism status (GMS), which was defined according to the World Health Organization 2006 criteria as normal glucose metabolism (NGM), impaired fasting glucose and impaired glucose tolerance (combined as prediabetes) or type 2 diabetes [31]. For safety reasons, participants using insulin or with a fasting glucose level >11.0 mmol/l (determined by finger prick) did not undergo the OGTT. These individuals were automatically classified as having diabetes. HbA_1c_, insulin and lipid profiles were measured in venous fasting blood samples, as previously described [24]. Insulin resistance was assessed by the HOMA-IR, which was calculated with the HOMA calculator version 2.2.3 for Windows (www.OCDEM.ox.ac.uk).

### Statistical analysis

Continuous data are presented as mean ± SD, or as median (interquartile range) in the case of non-normal distribution. Categorical data are presented as number (%).

First, to examine the effect of cross-sectional interaction between IHL content and the PRS for triglyceride clearance on serum triglycerides, we performed multivariable linear regression analysis as the primary analysis. IHL content, the PRS for triglyceride clearance and the interaction term IHL content × PRS for triglyceride clearance were used as exposures, and (log_10_) serum triglycerides was used as the main outcome. To obtain interpretable results, we back-transformed the regression coefficients, which should be interpreted as the fold change in serum triglycerides that is associated with a 1 unit increase in IHL content or PRS [14]. The following regression models were used for the analyses: model 1: crude model; model 2: with adjustment for age, sex, type 2 diabetes (because of the oversampling of type 2 diabetes in The Maastricht Study), MRI lag time (i.e. the time between the basic measurements and the MRI measurement of the liver, included as the interaction term ‘IHL content × MRI lag time’) and the first ten principal components of population stratification; model 3: with additional adjustment for lipid-modifying medication and alcohol intake.

Second, we classified the participants into six groups according to steatosis status (binary, yes/no) and PRS for triglyceride clearance (in tertiles, low/intermediate/high) to study the effect of interaction on risk of hypertriglyceridaemia in a multiple logistic regression analysis.

Several sensitivity analyses were performed. We repeated the analyses after: (1) stratification by sex; (2) stratification by type 2 diabetes status; (3) stratification by lipid modification; (4) replacement of IHL content by HOMA-IR.

All analyses were also repeated after replacing the PRS for triglyceride clearance by a PRS for triglyceride genes involved in ‘triglyceride production only’ (*n*=12), and a PRS involving nine triglyceride genes randomly selected from all 142 genes (ESM Methods, ESM Table 1). For the latter, we selected 500 sets of nine triglyceride genes at random from all genes, and then generated the mean value, a null distribution, of the interaction term IHL content × PRS and its CI using bootstrapping (*N*_bootstrapping_=5000).

Finally, we performed an exploratory analysis to study the impact of longitudinal interaction between IHL content/steatotic liver disease and PRS for triglyceride clearance on incident CVD risk using Cox proportional hazards regression analysis. The following models were applied: model 1: crude model; model 2: after adjustment for age, sex, type 2 diabetes, IHL content/steatosis × MRI lag time and the first ten principal components of population stratification; model 3: after additional adjustment for smoking status, alcohol intake, lipid-modifying medication, anti-hypertensive medication, systolic blood pressure and LDL-cholesterol.

The percentage of missing values for covariates was less than 1.1%, except for HOMA-IR (58.1% missing). The missing data were not imputed and were left as missing in all of the analyses. Statistical analyses were performed with the use of R statistical software version 4.0.1 (the R statistical software is available from https://cran.r-project.org/bin/windows/base/old/4.0.1/).

## Results

### General characteristics of the study population

Baseline characteristics of the overall population (*n*=3810) and stratified for serum triglycerides are shown in Table 2. Individuals included in this study appeared to have a somewhat better cardiometabolic profile than those who were excluded (ESM Table 2). The mean age of the study population was 60 ± 9 years, 48% were female, the median IHL content was 3.2% (interquartile range: 2.0–6.0%) and the median serum triglycerides level was 1.2 mmol/l (interquartile range: 0.9–1.7 mmol/l). The prevalence of type 2 diabetes, CVD, steatotic liver disease and hypertriglyceridaemia was 20%, 13%, 26% and 10%, respectively. Compared with participants in the lowest triglycerides tertile, those in the highest were older and more often male, and had a lower educational level and an unhealthier lifestyle. Participants in the highest triglycerides tertile were also characterised by a more adverse cardiometabolic profile, as reflected by lower HDL-cholesterol, and higher BMI, LDL-cholesterol, HbA_1c_, and systolic and diastolic blood pressure, and higher prevalence of prediabetes, type 2 diabetes, steatotic liver disease and CVD (Table 2). When the participants were stratified according to tertiles of PRS for triglyceride clearance, all the characteristics were comparable between different groups (ESM Table 3). Participants in the highest tertile had modestly higher serum triglyceride levels (1.2 mmol/l; interquartile range: 0.9–1.8) as compared with the lowest tertile (1.1 mmol/l; interquartile range: 0.8–1.5) (ESM Table 3).

**Table 2:** Baseline characteristics of the study population stratified according to serum triglycerides (*n*=3810)

Characteristic	Total (*n*=3810)	First tertile (*n*=1278)	Second tertile (*n*=1281)	Third tertile (*n*=1251)
Triglycerides, mmol/l	1.2 (0.9–1.7)	0.8 (0.6–0.9)	1.2 (1.1–1.3)	2.0 (1.7–2.5)
Age, years	60 ± 9	58 ± 9	60 ± 8	61 ± 8
Women, %	48	57	48	40
Education, % low/medium/high				
Low	32	27	31	38
Medium	28	28	29	27
High	40	45	40	35
Alcohol intake, g/day	9.1 (1.9–19.1)	8.6 (1.9–17.7)	9.1 (2.6–19.0)	9.8 (1.6–20.2)
Smoking status, % Never/Former/Current				
Never	39	43	39	33
Former	50	48	49	53
Current	11	9	12	14
BMI, kg/m^2^	26.5 ± 4.1	24.7 ± 3.3	26.4 ± 3.8	28.4 ± 4.2
OSBP, mmHg	133 ± 17	129 ± 17	134 ± 17	137 ± 17
ODBP, mmHg	75 ± 10	73 ± 10	76 ± 9	78 ± 10
Anti-hypertensive medication, %	33	22	33	45
Total cholesterol, mmol/l	5.3 ± 1.1	5.1 ± 1.0	5.3 ± 1.1	5.5 ± 1.2
HDL-cholesterol, mmol/l	1.6 ± 0.5	1.8 ± 0.5	1.6 ± 0.4	1.3 ± 0.3
LDL-cholesterol, mmol/l	3.1 ± 1.0	2.9 ± 0.8	3.2 ± 1.0	3.2 ± 1.1
Hypertriglyceridaemia, %	10	0	0	31
Lipid-modifying medication, %	28	19	28	39
HbA_1c_, mmol/mol	37 (34–41)	35 (33–39)	37 (34–41)	39 (35–44)
HbA_1c_, %	5.5 (5.3–5.9)	5.4 (5.2–5.7)	5.5 (5.3–5.9)	5.7 (5.4–6.2)
GMS, %, NGM/prediabetes/type 2 diabetes/other types of diabetes				
NGM	65	82	66	48
Prediabetes	14	8	14	21
Type 2 diabetes	20	9	19	31
Other types of diabetes	1	1	1	0
Glucose-lowering medication, %	15	8	15	22
HOMA-IR	1.3 (0.9–2.0)	1.0 (0.8–1.4)	1.3 (0.9–1.9)	1.8 (1.3–2.6)
History of CVD, %	13	10	14	14
IHL content, %	3.2 (2.0–6.0)	2.4 (1.5–3.8)	3.2 (2.0–5.5)	5.1 (3.0–9.8)
Steatotic liver disease, %	26	10	23	44

Data are shown as percentage, mean ± SD or median (interquartile range)

ODBP, office diastolic blood pressure; OSBP, office systolic blood pressure

### Effect of interaction between IHL content and PRS for triglyceride clearance on serum triglyceride levels

**Fig. 2:**
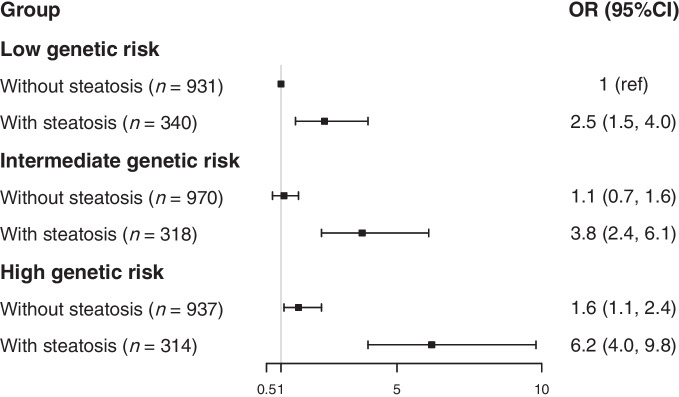
Effect of interaction between steatotic liver disease and PRS for triglyceride clearance on the risk of hypertriglyceridaemia (*n*=3810). Participants were divided into six groups according to steatosis status and genetic risk level. Individuals without steatotic liver disease and with low genetic risk were used as the reference group. The analyses were adjusted for age, sex, type 2 diabetes status, MRI lag time × steatosis, first ten principal components of population stratification, lipid-modifying medication and alcohol intake. Ref, reference

Both IHL content and PRS for triglyceride clearance were associated with higher serum triglycerides after full adjustment (β 1.011; 95% CI 1.009, 1.013; and β 1.213; 95% CI 1.151, 1.279, respectively). We found an effect of interaction between IHL content and the PRS for triglyceride clearance on serum triglycerides in the fully adjusted model (*p* for interaction: 0.005; Table 3). Additional adjustment for BMI did not alter the results (*p* for interaction: 0.012; data not shown). To facilitate clinical translation, we subsequently repeated the analyses with the exposure and outcome variables expressed as categorical variables. A high PRS for triglyceride clearance only modestly increased the risk for hypertriglyceridaemia in participants without steatotic liver disease (OR 1.618; 95% CI 1.110, 2.380; Fig. 2). Of interest, this risk was substantially higher when steatotic liver disease was also present (OR 6.196; 95% CI 3.966, 9.768; Fig. 2).

**Table 3:** Effect of interaction between IHL content and PRS for triglyceride clearance on serum triglycerides (*n*=3810)

Variable	β (95% CI)		
	Model 1	Model 2	Model 3
IHL content	1.008 (1.004, 1.013)	1.005 (1.001, 1.009)	1.005 (1.001, 1.010)
PRS for triglyceride clearance	1.149 (1.072, 1.232)	1.137 (1.062, 1.218)	1.135 (1.060, 1.216)
Interaction term	1.010 (1.001, 1.019)	1.013 (1.004, 1.022)	1.013 (1.004, 1.022)
*p* value for interaction	0.031	0.004	0.005

Model 1: crude model

Model 2: as model 1 with additional adjustment for age, sex, type 2 diabetes, IHL content × MRI lag time and the first ten principal components of population stratification

Model 3: as model 2 with additional adjustment for lipid-modifying medication and alcohol intake

β values are all expressed as fold change in serum triglycerides per unit increase in exposure

When the analyses were stratified by sex, type 2 diabetes status and lipid-lowering medication, the interaction between IHL content and the PRS for triglyceride clearance was more pronounced in women (ESM Table 4, ESM Fig. 3), did not differ between participants with or without type 2 diabetes (ESM Table 5, ESM Fig. 4) and was more pronounced in participants without lipid-lowering therapy (ESM Table 6, ESM Fig. 5). Replacement of IHL content by HOMA-IR (*n*=1.594) did not result in a statistically significant interaction (ESM Table 7).

When the PRS for triglyceride clearance was replaced by either the PRS for triglyceride production or the PRS for nine randomly selected triglyceride genes, their associations with serum triglycerides were comparable to the PRS for triglyceride clearance (β 1.248; 95% CI 1.173, 1.328 for the PRS for triglyceride production; β 1.211; 95% CI 1.196, 1.227 for the PRS for nine randomly selected triglyceride genes; vs β 1.213; 95% CI 1.151, 1.279 PRS for triglyceride clearance). However, replacement of the PRS for triglyceride clearance by the PRS for triglyceride production did not show an effect of interaction with IHL content on serum triglyceride levels after full adjustment (*p* value for interaction: 0.993, *n*=3769 participants; ESM Table 8), and nor did replacement by a PRS involving nine randomly selected triglyceride genes (*p* value for interaction: 0.192; ESM Table 9).

### Effect of interaction between IHL content and PRS for triglyceride clearance on incident CVD risk

During 24,390 person-years of follow-up, 318 of 3127 individuals developed a CVD event, corresponding with an incidence rate of 13 cases per 1000 person-years.

**Fig. 3:**
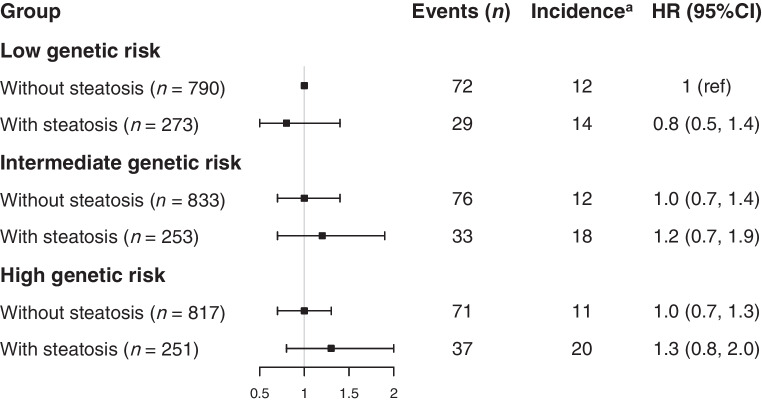
Effect of interaction between steatotic liver disease and PRS for triglyceride clearance on incident CVD (*n*=3217). Participants were divided into six groups according to steatosis status and genetic risk level. Individuals without steatotic liver disease and with low genetic risk were used as the reference group. The analyses were adjusted for age, sex, type 2 diabetes status, steatosis × MRI lag time, first ten principal components of population stratification, smoking status, alcohol intake, lipid-modifying medication, anti-hypertensive medication, systolic blood pressure and LDL-cholesterol. ^a^Incidence per 1000 person-years. Ref, reference

After full adjustment, we observed a similar effect of interaction between IHL content and PRS for triglyceride clearance on incident CVD in the fully adjusted model, albeit not statistically significant (*p* for interaction: 0.078; ESM Table 10). Similar outcomes were observed when the exposure and outcome variables were expressed as categorical variables (Fig. 3).

## Discussion

In this study, we found an effect of interaction between IHL content and PRS for triglyceride clearance on serum triglycerides. While a high PRS was associated with only modestly higher risk of hypertriglyceridaemia in individuals without steatotic liver disease (OR 1.618; 95% CI 1.110, 2.380), a substantially stronger association was observed in those individuals with steatotic liver disease (OR 6.196; 95% CI 3.966, 9.768). A similar modifying non-significant effect was observed when incident CVD was studied as an outcome variable.

Twin studies have shown that the heritability of serum triglycerides is high (~60%) [32]. Although hypertriglyceridaemia is a highly polygenic trait [9], to date only 11% of the variance in serum triglyceride concentrations has been explained by common genetic variants [33]. Recent studies have shown that this missing heritability is partly explained by gene–environment interactions [34–36]. To date, only a few studies have focused on gene–environment interactions in explaining the variance in serum triglycerides. Esteve-Luque and colleagues reported that a PRS for triglycerides was modified by BMI in 276 participants [37]. Our results extend and refine these findings. We observed a strong interaction between our PRS for triglyceride clearance and steatotic liver disease. This is explained by careful selection of gene variants. While others previously constructed a PRS for triglycerides in an agnostic manner by taking all GWAS loci [38–40], we reasoned that steatosis-driven VLDL overproduction [21] will have an amplifying effect on the risk of hypertriglyceridaemia when there is a specific genetic impairment of triglyceride clearance. Indeed, when we replaced the PRS for triglyceride clearance by a PRS for serum triglyceride genes with a known biological function other than triglyceride clearance or a random selection of all genes associated with serum triglycerides, we did not observe such an interaction.

The consequences of our study are several-fold. First, it provides more insight into the complex pathogenesis of familial dyslipidaemias. We have previously shown that the prevalence of steatotic liver disease is high in individuals with familial combined hyperlipidaemia (FCHL) [41], in particular among individuals with the hypertriglyceridaemic phenotype (~66%) [42]. Moreover, gene variants that affect triglyceride clearance, such as *LPL*, *LIPC* and *GALNT2*, are enriched in FCHL pedigrees [43]. The interaction between these genes and steatotic liver disease, as observed in this study, likely contributes to the pathogenesis of hypertriglyceridaemia in this highly prevalent disorder (~1%) [43]. Second, the observed interaction allows precision medicine. The use of nine gene variants, each with limited individual clinical impact, has the potential to identify individuals who have a high risk of developing hypertriglyceridaemia (and subsequently of CVD) if they develop steatotic liver disease, a condition that is highly prevalent in individuals with obesity and/or type 2 diabetes. While further validation is needed, this insight could enhance our understanding of the pathophysiology of hypertriglyceridaemia and CVD. Based on the great difference in risk of hypertriglyceridaemia in high genetic risk carriers with and without steatotic liver disease (1.618; 95% CI 1.110, 2.380; vs OR 6.196; 95% CI 3.966, 9.768), it is anticipated that treatment of steatotic liver disease will substantially reduce the risk of hypertriglyceridaemia (and consequently CVD) in these people. The clinical relevance will require further studies.

This study has several strengths. First, we used data from a large population-based cohort that was extensively phenotyped and genotyped using state-of-the-art methods. This allowed for an accurate estimation of IHL content, serum triglyceride concentrations and genotyping data. Second, by analysing both continuous, i.e. serum triglyceride concentrations and IHL content, and categorical, i.e. hypertriglyceridaemia and steatotic liver disease, traits our results are relevant and robust. Third, we used longitudinal data for incident CVD, ruling out reverse causality. Our study also has limitations. First, although this study was sufficiently powered for the primary outcome, it probably was not for incident CVD. Second, the findings of our study need to be validated in another cohort, ideally from different ancestry. Third, the GWAS we used for the construction of the PRS included individuals of various ethnicities [28]. Notably, the majority (~70%) were of European ancestry which was similar to the participants in our study (~99% of European ancestry). Fourth, while participants were recruited from the general population, there was an oversampling of type 2 diabetes. We, therefore, corrected for type 2 diabetes in all analyses and additionally performed stratified analyses, which did not show differences in the interaction of interest between participants with and without type 2 diabetes. Last, participants included in the analyses were characterised by a more favourable cardiovascular risk profile than those who were excluded due to missing data. These differences were, however, minor.

In conclusion, this population-based study demonstrates that a PRS for triglyceride clearance interacts with steatotic liver disease to impact the risk of hypertriglyceridaemia. This gene–environment interaction might contribute to more personalised treatment approaches, which require further exploration in future studies.

## Supplementary Information

Below is the link to the electronic supplementary material.

Supplementary file1 (PDF 989 KB)

Supplementary file2 (XLSX 76 KB)

## Data Availability

Data are available from The Maastricht Study for any researcher who meets the criteria for access to confidential data; the corresponding author may be contacted to request data.

## Abbreviations

FCHL: Familial combined hyperlipidaemia
GMS: Glucose metabolism status
GWAS: Genome-wide association study
^1^H-MRS: Proton magnetic resonance spectroscopy
IHL: Intrahepatic lipid
NAFLD: Non-alcoholic fatty liver disease
PRS: Polygenic risk score
