# Supplementary material for: Steatotic liver disease interacts with a polygenic risk score for triglyceride clearance to impact the risk of hypertriglyceridaemia: The Maastricht Study
Source: Diabetologia. 2025 Jun 27;68(10):2217–26. doi: 10.1007/s00125-025-06479-3 (PMC12423141; doi:10.1007/s00125-025-06479-3)
Supplement: Supplementary file 1 — Supplementary file1 (PDF 989 KB) [file 125_2025_6479_MOESM1_ESM.pdf]

## Electronic Supplementary Material

### Methods

### The role of triglyceride genes in triglyceride metabolism

We retrieved genes associated with serum triglycerides from a large-scale genome-wide association study (GWAS) (1). This GWAS reported 151 genes associated with serum triglycerides in total (Supplementary Table 3 and 7 in the GWAS (1)). Among them, 9 SNPs were excluded as they are monoallelic in Europeans. The roles of the remaining 142 genes in triglyceride metabolism were determined by PubMed and Genecards search. As the triacylglycerol-rich very-low-density lipoprotein (VLDL) contributes to most of the serum triglyceride concentrations, we searched the literature on PubMed with the keywords 'gene name' + 'VLDL' manually, then the genes were classified into 5 categories based on the results on PubMed: 1) involved in triglyceride clearance only (n = 9); 2) involved in triglyceride production only (n = 12); 3) involved in triglyceride production *and* clearance (n = 5); 4) uncertain function (although there were results on PubMed based on the keywords, the roles of these genes were uncertain according to the literature, n = 8); and 5) no results (n = 108).

### Effect size of the genes

The effect size (beta value) for each gene in the calculation of polygenic risk score (PRS) was retrieved from Supplementary Table 3 and Supplementary 7 in the cited GWAS (1). Since that GWAS only validated the novel genes in the replication analysis, not all the genes have the beta values from the meta-GWAS. Therefore, we used the beta values from the Million Veteran Program (MVP) cohort in the discovery analysis.

### Reference


1. Klarin D, Damrauer SM, Cho K, et al. Genetics of blood lipids among ~300,000 multi-ethnic participants of the Million Veteran Program. Nat Genet 2018;50:1514-1523.

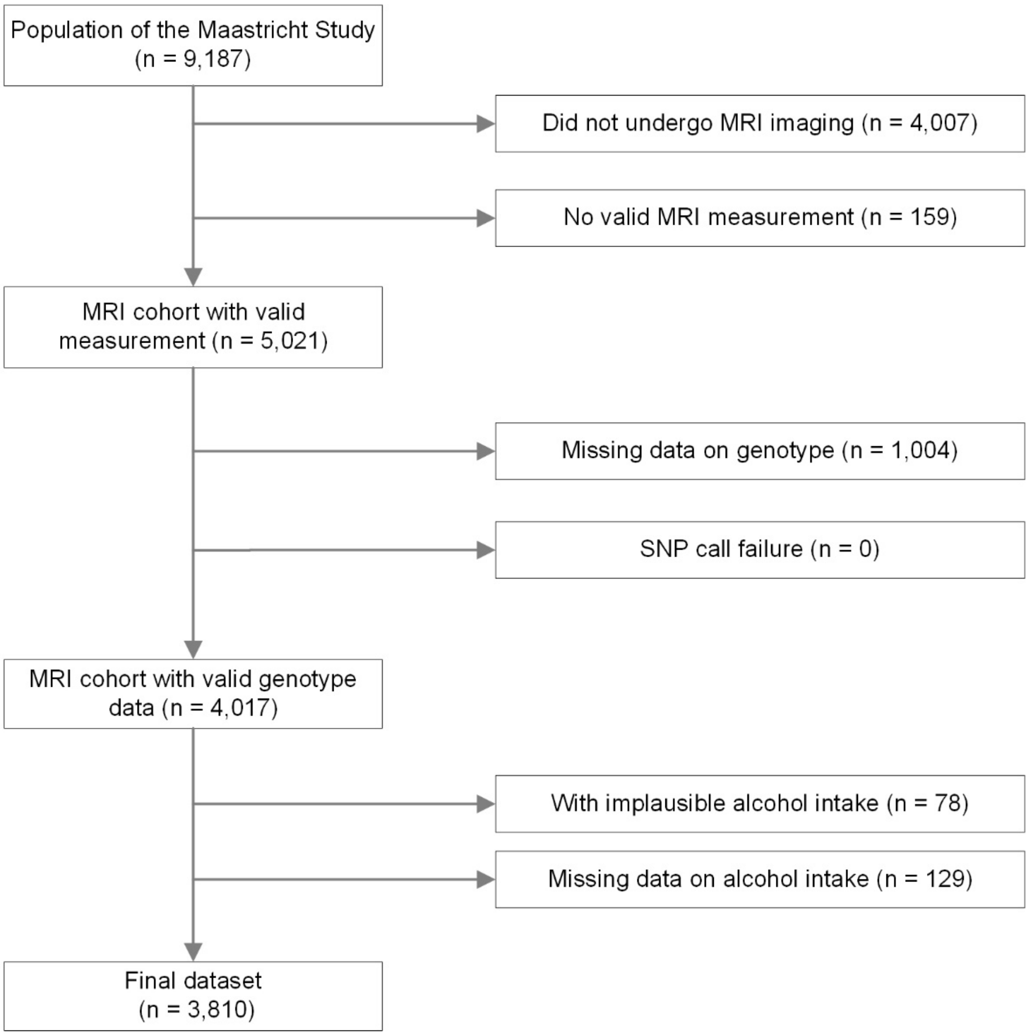


**ESM Fig.1 Flowchart of the inclusion and exclusion of the study population.**

Abbreviations: MRI, magnetic resonance imaging; SNP, single nucleotide polymorphism

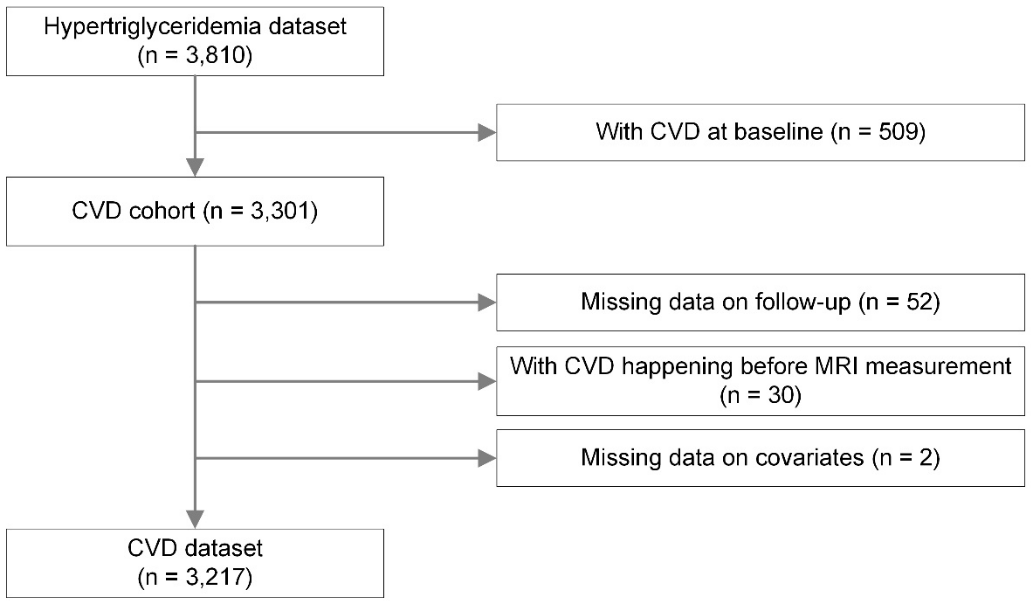


**ESM Fig.2 Flowchart of the inclusion and exclusion of the study population in the CVD dataset.**

Abbreviations: CVD, Cardiovascular disease; MRI, Magnetic resonance imaging.

A)

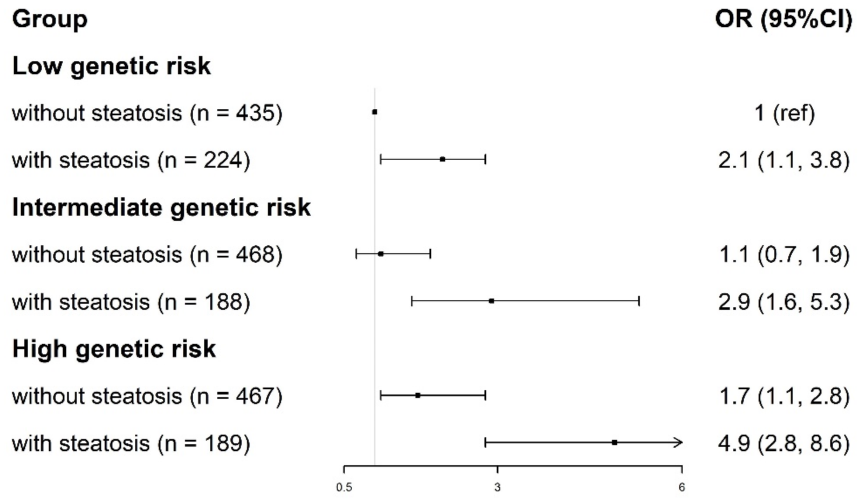


B)

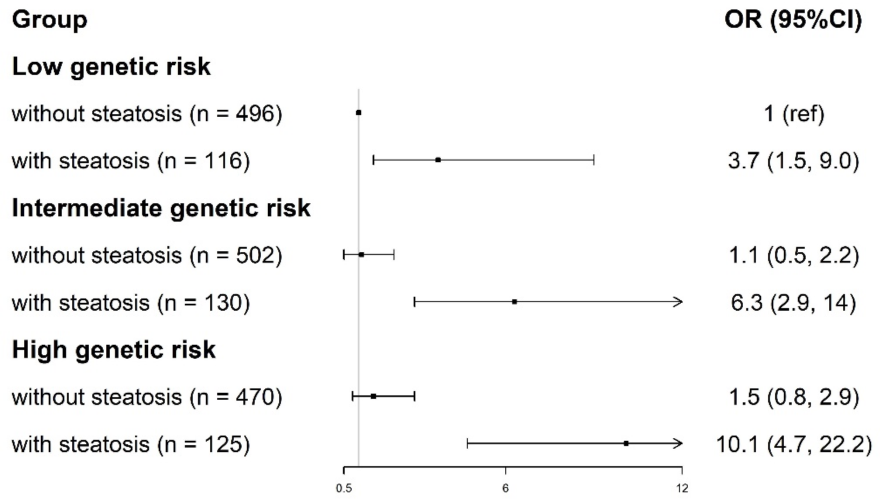


**ESM Fig.3 Interaction between steatosis and polygenic risk score for triglyceride clearance on the risk of hypertriglyceridaemia stratified by sex.**

Panel A) showed the result in men (n = 1,971); Panel B) showed the result in women (n = 1,839). The result was with adjustment of age, type 2 diabetes status, steatosis * MRI lag time, first ten principal components of population stratification, lipid-modifying medication and alcohol intake. Abbreviations: CI, confidence interval; OR, odds ratio; Ref, reference.

A)

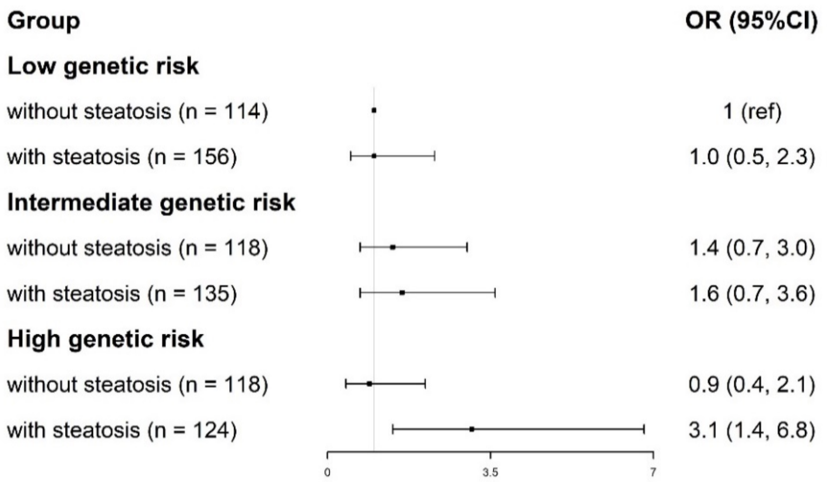


B)

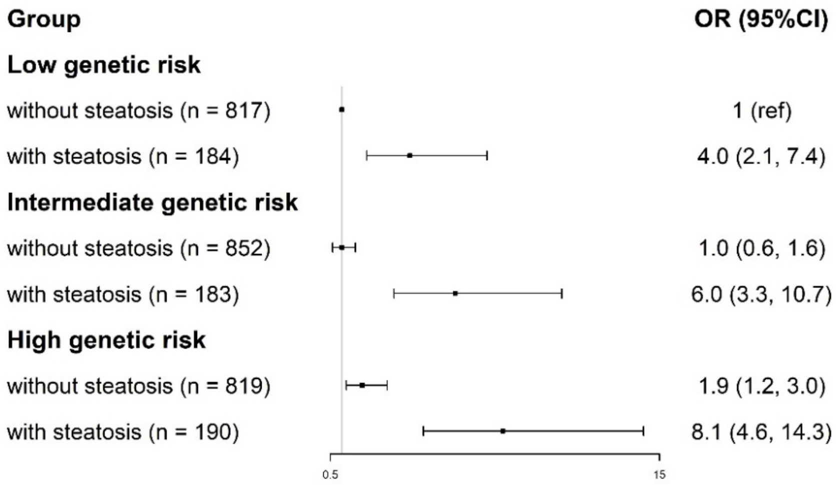


**ESM Fig.4 Interaction between steatosis and polygenic risk score for triglyceride clearance on the risk of hypertriglyceridaemia stratified by T2D status.**

Panel A) showed the result in participants with T2D (n = 765); Panel B) showed the result in participants without T2D (n = 3,045).

The result was with adjustment of age, sex, steatosis * MRI lag time, first ten principal components of population stratification, lipid-modifying medication and alcohol intake.

Abbreviations: CI, confidence interval; OR, odds ratio; Ref, reference; T2D, type 2 diabetes.

**A)**

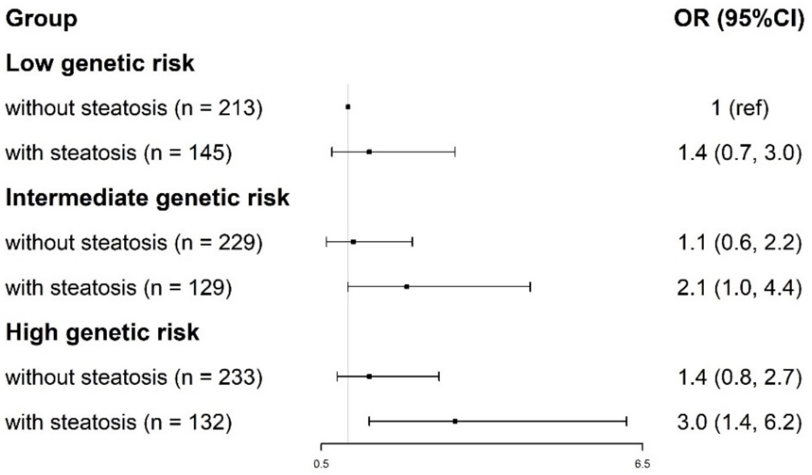


**B)**

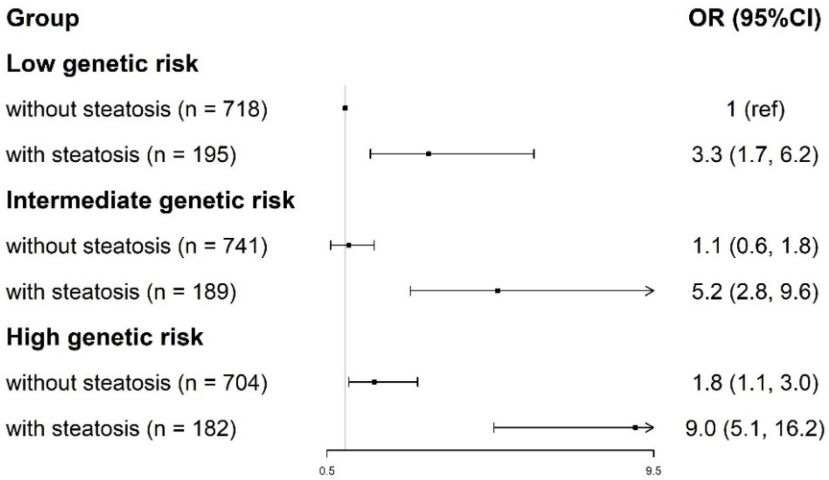


**ESM Fig.5 Interaction between steatosis and polygenic risk score for triglyceride clearance on the risk of hypertriglyceridaemia stratified by lipid-modifying medication.**

Panel A) showed the result in participants with lipid-modification (n = 1,081); Panel B) showed the result in participants without lipid modification (n = 2,729).

The result was with adjustment of age, sex, type 2 diabetes status, steatosis * MRI lag time, first ten principal components of population stratification and alcohol intake.

Abbreviations: CI, confidence interval; OR, odds ratio; Ref, reference.
